# Governance for net zero project evaluation: Experiences from UK local authorities

**DOI:** 10.1007/s12053-026-10422-9

**Published:** 2026-02-24

**Authors:** Daniel Kerr, Andrew Reeves, John Rowlatt

**Affiliations:** Institute for Sustainable Futures, De Montfort University, Leicester, United Kingdom

**Keywords:** Net zero, United Kingdom, Local government, Local area energy planning, Planning

## Abstract

The United Kingdom has committed, through a legally-binding target, to achieve net zero emissions in the UK economy by 2050. The UK government envisages local authorities as being critical actors in achieving net zero. However, no coherent national guidance regarding net zero implementation exists for local governments. In the context of evolving national- and regional- scale energy planning, with the formation of Great British Energy, the National Energy System Operator and other bodies, local authorities will have an increasing role in energy planning. However, funding environments, human resources and technical capacity remain constrained. Strong governance mechanisms will be required to manage the socio-technical transition to a net zero future in the UK, and to engage with multiple actors across spatial and political scales. This article draws on research examining the challenges that UK local authorities (at County Council, District Council and Combined Authority scales) are having with access to and analysis of data for meaningful evaluation of current projects and future project planning. Through semi-structured interviews with 19 separate actors from UK local authorities, national bodies and consultancies working in net zero with UK local governments, we: • Examine the evaluation gap in carbon reduction, project costs and progress to net zero. • Consider the effect that an uncoordinated national policy framework for net zero is having on the ability for local authorities to effectively evaluate future project development routes. • Discuss how the increasingly adopted Local Area Energy Planning methodology is affecting local net zero policy and spatial planning, and what governance frameworks are needed to ensure that a net zero project is well-facilitated, timely and accelerated. Our findings highlight principles for how governance for monitoring and evaluation of net zero projects is a necessary foundation for assessing the success or failure of interventions, having relevance in the UK and beyond.

**Supplementary Information:**

The online version contains supplementary material available at 10.1007/s12053-026-10422-9.

## Introduction

Local authorities have taken a number of roles in recent years in relation to climate change mitigation and adaptation, and the work towards net zero in the UK. Various approaches have been trialled and developed across the four devolved nations of the UK in response to the challenge of climate change: Local Heat and Energy Efficiency Strategies (LHEES) development in Scotland has complemented the emergence of Local Area Energy Plans (LAEPs), which have previously been a statutory obligation in Wales, and are developing in prominence in English and Northern Irish local authorities. However, progress towards the recommendations made in LAEPs has been fragmented and slow, and with nationally and locally defined targets for 2030 and 2050, the development and deployment of net zero projects needs to accelerate. Recent developments at a national and regional scale, with the formation of Great British Energy as a national-scale project finance body and the upcoming Regional Energy Strategic Plans, mean that governance at a local level needs to be responsive, and communicate to higher and lower spatial scales in an effective manner.

This paper investigates the governance of net zero and LAEPs in English local authorities, being unitary authorities, county/district and borough (“two-tier”) authorities and combined authorities.^1^Through a series of 19 semi-structured interviews with local authority officers, consultants and third-sector organisations, we investigate the challenges of governing the net zero system in English local authorities, innovative approaches adopted by trailblazer local authorities, and opportunities for the enhancement of governance structures and practices, as well as the absence of post-project evaluation and institutional learning observed in net zero project development and deployment. Through this analysis, we examine how project development and project delivery is compromised through the siloed nature of staff experience in local authorities, how learning opportunities are missed due to these capacity issues, and governance options for accelerating net zero project delivery through innovative governance across scales.

## Literature review & context

The UK government envisages local authorities as being critical actors in achieving net zero, and place-based action on climate change has become more prominent in the academic literature on net zero in recent years. There are a number of roles that local governments can take as governance bodies in relation to net zero, which are represented in the literature in four main categories:

Strategic direction at a local and regional level – local governments can take different levels of responsibility for defining strategy for net zero conceptualisation and intervention. Local governments can take roles as influencers, coordinators and sometimes implementers themselves. At a high level, Den Uyl and Russel (2017) recommend local governments act as responsible, accountable bodies which own the “problem” of net zero, and act as central reporting points for evaluation. Marsden and Rye (2010) in their assessment of transport governance recommend clear roles for market actors and clear accountability structures for local governments.

Regulation and enforcement – local governments can own the role of regulator for net zero interventions. Regulation covers standards of action, but also expected actions of market actors. Poulter and Bolton (2021) suggest regulatory frameworks need simplicity and clarity of purpose to avoid “gaming” of systems. Busch et al. (2021) recommend co-creation of regulatory frameworks with the proposed regulated actors to ensure regulations are realistic and fit for purpose. Devolution remains a barrier however: Sugar and Webb (2022) highlight the limited powers of local governments and the need for further devolution of powers for net zero interventions to be implemented.

Facilitating collaboration – a critical role of local governments for governing the net zero transition will be collaborative structures and facilitating collaborative decision making between the diverse stakeholder groups involved in net zero. The lack of collaboration between scales of governance is a consistent barrier from the literature: Bedford et al. (2023) cite the limited collaboration between scales of local governments to regional governments, and the “limited ability to integrate policies locally” as a key barrier. Berthod et al. (2022) address collaborative procedures between local governments and implementation partners, and the need for equality in powers between partners in implementation projects, and not letting embedded power structures dominate discussions. Carr-Whitworth et al. (2023) recommend incentivising collaboration and rewarding impact as an aspect of net zero governance. Finally, Linton et al. (2020) in their assessment of governance for deep decarbonisation at a local level recommend facilitation of cross-sector collaboration as a role of local governments.

Responsibility – Carr-Whitworth et al. (2023) list clarifying governance structures, expectations and responsibilities as one of their twelve key conditions for success in delivering net zero. Local governments can have a role as the responsible body for net zero actions, but the need for greater collaboration across scales and multi-level governance is noted. Local governments need more resources, funding and capacity to act as local-level implementers. Tingey and Webb (2020) also discuss the need for clearer devolution of responsibilities from national to local, and for different levels of local governments to take responsibility for net zero interventions at the governance scale, as well as implementation.

Den Uyl and Russel (2017) advocate for accountable bodies for climate adaptation in public administration, and highlight the challenges of capacity and the need for resource to deliver interventions. Marsden and Rye (2010) argue for clear roles for stakeholders and transparent accountability structures in transport governance for climate change. Willis et al. (2022) in their assessment of deliberative democracy for addressing the climate crisis advocate for clear stakeholder engagement processes and strong coordination between the deliberative mini-publics (DMPs) they describe, such as Climate Assembly UK, and decision-making bodies. Linton et al. (2020) describe various modes of governance (self-governance, governing through enabling and others) and point to cross- and inter-sectoral communication as vital to the process.

Tingey and Webb (2020) synthesise a number of these challenges in their paper on valuation practices for energy projects in UK local authorities: while the Best Value, business case and public procurement models are suggested to provide an appropriate evaluation of the value of local investment options, the authors found that the parameterisation of value in local governments often excluded co-benefits such as social value. Lampard et al. (2023) in their review of health-related impacts of climate change and their assessment in local authorities found that the availability of evidence to inform policy-making in local authorities is important, and crucial in the areas of community involvement in local-level action on the health impacts of climate change, as well as the economic costs of climate action. They also noted that solely providing better evidence to policy-makers is not sufficient to enable greater climate action.

## Methodology

Our primary research question was “what are the challenges that UK local authorities (at County Council, District Council and Combined Authority scales) with access to and analysis of data for meaningful evaluation of current projects and future project planning”. Our objectives were to:

- Examine the evaluation gap in carbon reduction, project costs and progress to net zero.
- Consider the effect that an uncoordinated national policy framework for net zero is having on the ability for local authorities to effectively evaluate future project development routes.
- Discuss how the increasingly adopted LAEP methodology is affecting local net zero policy and spatial planning, and what governance frameworks are needed to ensure that a net zero project is well-facilitated, timely and accelerated.

To achieve the above objectives, semi-structured interviews were selected as an appropriate methodology for data collection. Semi-structured interviews allow for an overarching structure for discussions focused on pre-prepared questions, while allowing for the flexibility to interrogate topics of interest that come up during the interview responses.

For participants, our focus was on interviewing contexts that were similar to the Leicestershire Collaborate to Accelerate Net Zero (LCAN phase 2) project’s focus area of Leicestershire, being a two-tier local authority structure of a County Council with subsidiary District Councils, integrating a unitary authority (City of Leicester) into climate change and LAEP planning processes. The inclusion criteria were Council officers or elected members working on LAEPs or climate change topics, or areas of related work such as nature recovery strategies, or consultants/business representatives involved in the delivery of projects under Council climate change programmes. Exclusion criteria were officers or elected representatives not working on climate change projects or related topics, or officers/consultants/businesses working in related areas that had no involvement with climate change project delivery or planning. The interview schedule is available as supplementary material. Participants were initially approached via email in all cases. Of the approached participants, one refused to participate due to time constraints. The interviews were conducted by 2 male PhD researchers employed as Research Fellows, with qualitative research experience from PhD research and other research projects. No prior relationship between interviewers and interviewees was established, although many interviewees were working on projects funded under the same funding call as this project. Data saturation was discussed by the interviewers, and efforts made during interviewee selection to avoid similar contexts. The interviews for this project were conducted over the period of July to December 2024. Each interview was scheduled for an hour. Interviews were conducted using Microsoft Teams, and transcripts and recordings of the interviews were kept for further analysis. Transcripts were not returned to participants, but were correlated against recordings by the interviewers. Interviewees were introduced to the project and the goals of the research prior at the start of the interview, as well as the background of the interviewers. No other non-participants were present during the interviews (Tong et al., 2007).

In total, 19 items have been considered in this analysis. 17 of these were semi-structured interviews using the question set outlined above. A further two interviews were included in the thematic analysis: a semi-structured interview with a national-level consultancy involved in LAEP development, and a transcript of a roundtable of investors involved with financing in local governments. These were included to provide more insight into the national-scale processes of LAEP governance and financing, to complement the local-scale data collection. In total, six of these interviews were with unitary authority officers, four with county council officers, two with combined authority officers, five with consultants working with local governments, and two with financiers.

To analyse the data from the interviews, thematic analysis was selected as a methodology to take a systemic look over the corpus of interview data and draw out relevant themes to meet the aims and objectives of the research. Thematic analysis (Braun and Clarke, 2006) is a qualitative analysis methodology based on defining multiple “themes”, either prior to the analysis for results that could be expected, or during the analysis for emerging topics of discussion. Nvivo was selected as the software package for analysis of the interviews. Interview transcripts were cleaned for errors and loaded into Nvivo, then coded using in-built tools for this analysis. 9 top-level themes were defined prior to the analysis, based on the results of the literature review and phase 1 of the LCAN project. These were: Accountability and Ownership, Resource and Capacity, Communications and Information, Stakeholder Engagement, Engagement with Neighbourhoods and Communities, Coordination and Collaboration, Future-Readiness, Fair, Equitable and Inclusive, and Leadership and Agenda-Setting. Two top-level themes were added during the analysis, being LAEP Information, and Project Delivery. The sub-codes for top-level themes were defined prior to the analysis based on the literature review and phase 1 of the LCAN project, aside from “Behaviour Change” as a sub-code of Future-Readiness, which was added in response to the interview content, and the themes added during analysis, which were sub-coded as part of the analysis. A table of codes, sub-codes and definitions is available as supplementary material.

Coding of the corpus was performed on a paragraph level where appropriate, and a sentence level if paragraphs contained multiple themes. Five of the interviews were subject to an inter-coder reliability analysis following the main coding by the lead author, which displayed good alignment with the main coding.

To further analyse this data, specific corpus searches for the term “evaluation” and stemmed words (“evaluate”, “evaluating” etc.) and the term “reflection” and stemmed words were performed. For “evaluation” and stems, 9 mentions across 8 separate interviews were found. For “reflection” and stems, 18 mentions from 10 interviews were found, however 10 of these mentions used other senses of the word “reflect” and were hence excluded from further analysis.

## Results

**Fig. 1:**
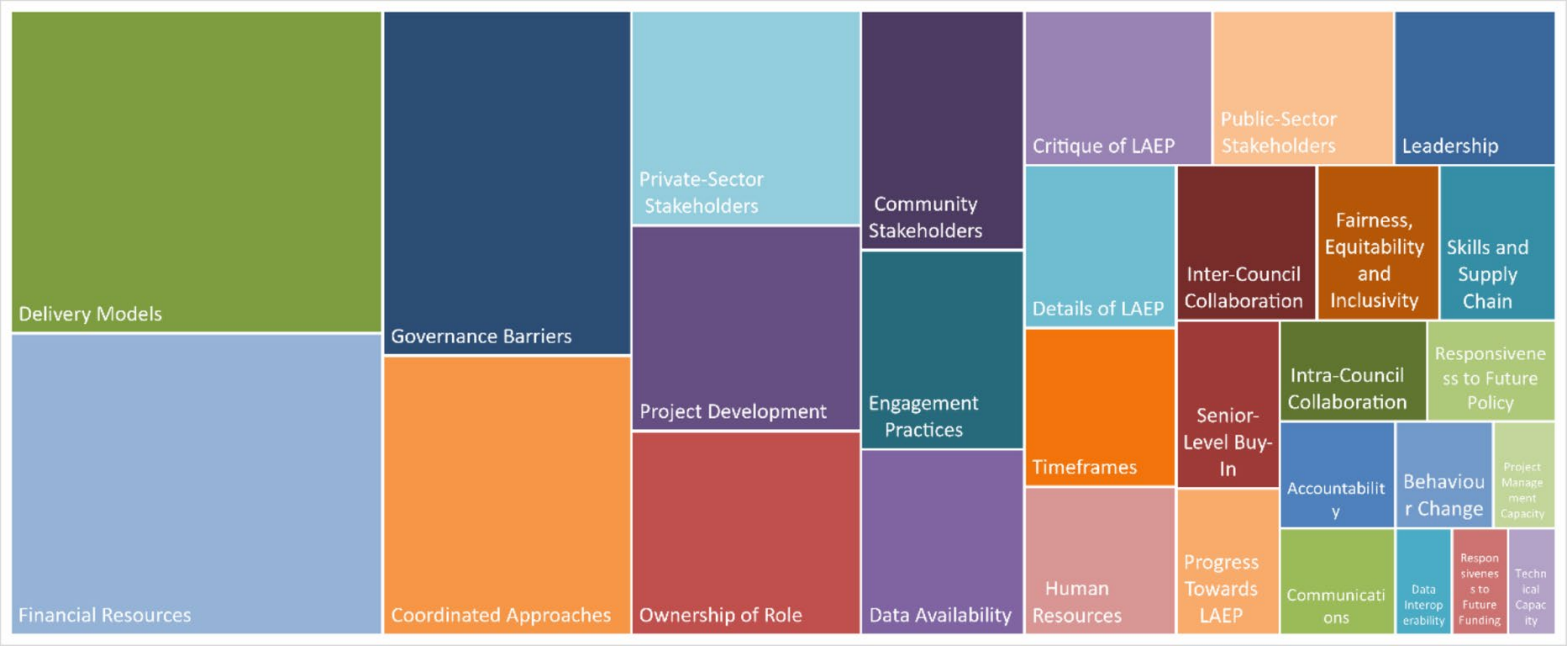
Hierarchy chart of most commonly-coded items

**Fig. 2:**
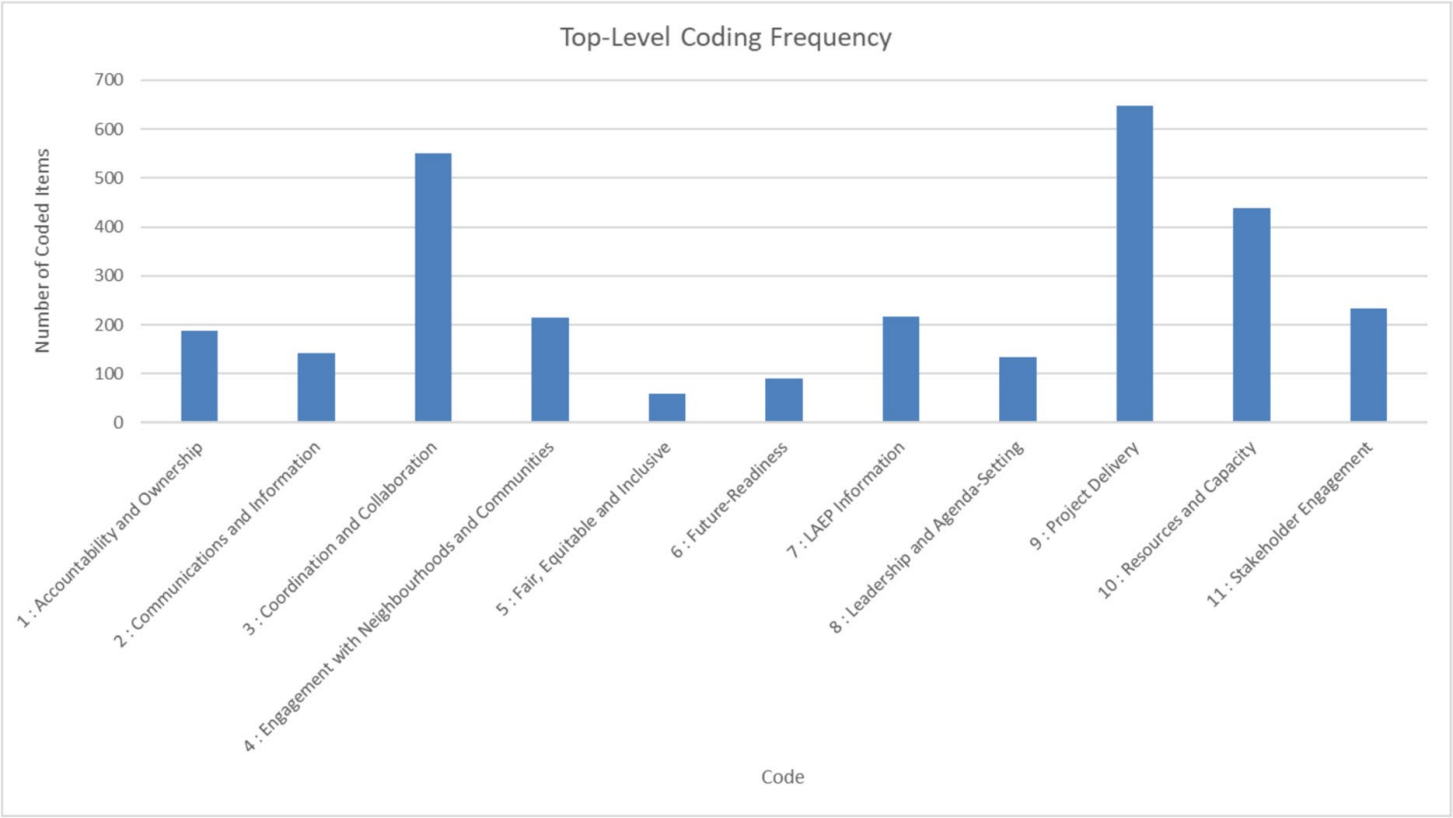
Frequency of eleven themes from Nvivo analysis

**Fig. 3:**
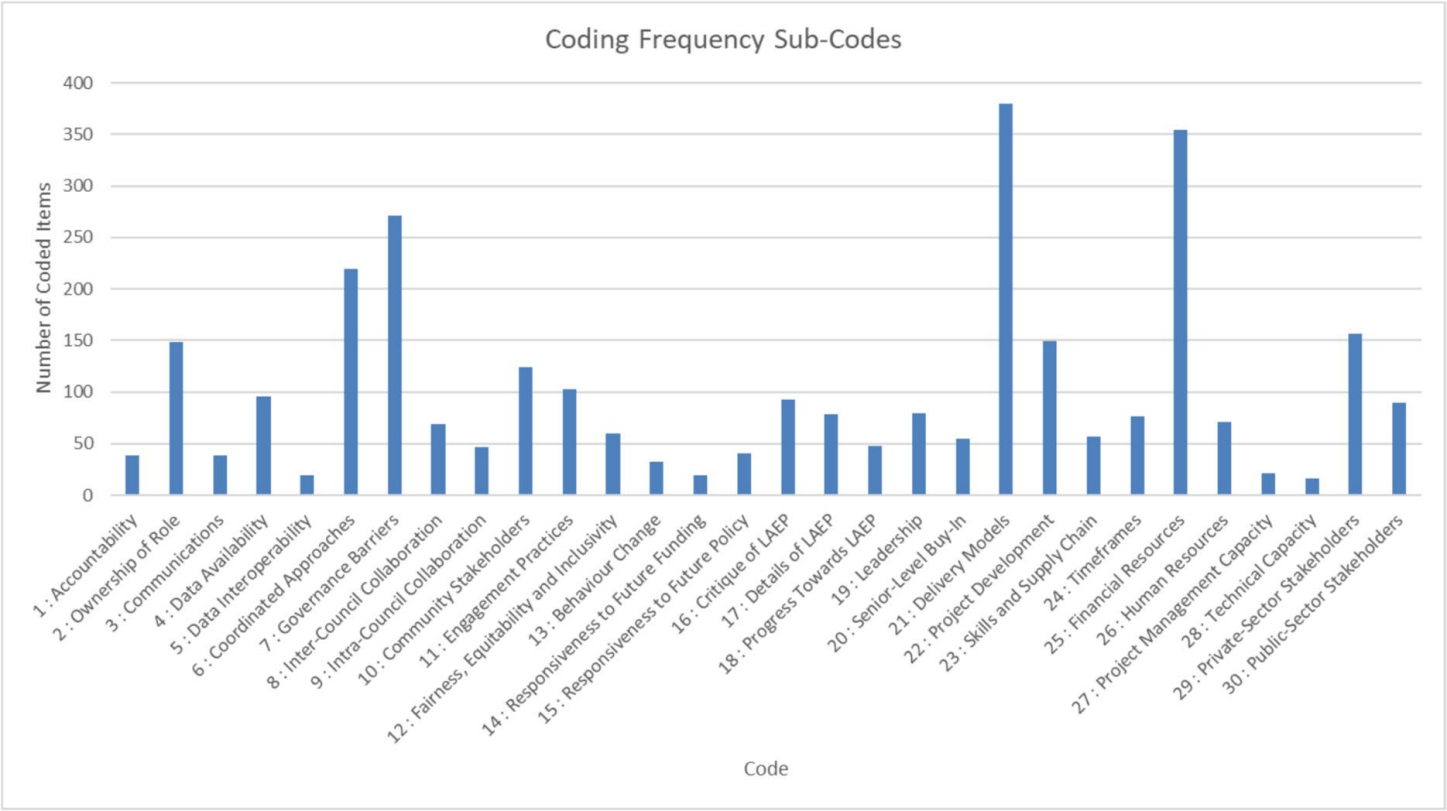
Frequency of thirty sub-codes identified under the themes from Nvivo analysis

Figure 1 shows the hierarchy chart of the most commonly-coded items, arranged in descending order of frequency in the corpus:

Figure 2 shows the overall frequency of each of the eleven top-level themes:

Figure 3 shows the frequency of the thirty individual sub-codes that comprise the top-level codes:

Two specific sub-codes are most relevant to this analysis, being “data availability” and “data interoperability”, representing interview data discussing the availability of quantitative or qualitative data for net zero project development and delivery, and the ease of integrating different data sources from within or without local authorities to form a combined set for assessment. “Data Availability” was represented by 96 references in the corpus, with “Data Interoperability” being represented by 19 references. These themes were selected due to their relevance to the topic of evaluation and evaluation practice.

Regarding the availability of data, interviewees highlighted a number of ways in which data access and presence can affect project development, delivery and evaluation. Coordination of data across authorities was cited as a blocker to effective action: tools being developed under the ongoing Local Energy Net Zero Accelerator programme, the OnePlanet platform and the Net Zero Go platform were highlighted as attempts to address this challenge.^2^Specific challenges with LAEPs were also mentioned: a lack of access to the data used to produce a LAEP post-delivery was cited as a hindrance to delivering projects. The value of having an evidence base for decarbonisation, which interviewees defined as a quantitative evidence base in most instances, as well as an indication of overall net zero adaptation costs, were cited as vital engagement tools. However, in many instances, maintenance of data was also brought up as a challenge: local authorities may be given access to a variety of data sets, but are not responsible for maintaining their accuracy, leading to a perception that data is commonly out-of-date at point of use.

*“Everyone wants to have a report, but that's not the key outcome because the report is out of date as soon as it's published.” – Officer, Tier 1 Local Authority*

Many organisations were described as collecting data in a piecemeal fashion for individual projects, with limited efforts to collate this into a more comprehensive data set. LAEPs were commonly seen as an opportunity to aggregate disparate data into a single resource for further use.

In terms of data interoperability, the aforementioned platforms are making an effort to address this challenge, however internal data governance in local authorities was seen as a barrier to more effective collaboration, and accelerated project delivery. One interviewee described an “excessive nervousness” around data security, even for data which was not perceived to be sensitive to policies such as the General Data Protection Regulation (GDPR) (Officer, Unitary Authority). The challenges of coordinating multiple, independently-developed datasets across different local authorities into a cohesive whole for collaboration were also mentioned, alongside the fact that this work is often a project in itself, subject to capacity constraints in terms of financial and human resources.

*“I think there's, you know, there's issues with the non-domestic sector, there's issues with EPCs, there's issues with data update and maintenance, there's issues with linked data, so people just creating their own dataset for their own interests that might be the same or not the same” – Officer, Scottish Council*

There was a desire from interviewees for policy to be more evidence-linked to quantitative or qualitative data, and for more centralised, digitally-enabled platforms to aggregate this data and enable access for different local authorities.

Further analysis of the corpus of interview data was performed on the terms “evaluation” and stems, and “reflection” and stems. These terms were selected to capture the specific opinions of the interview participants regarding evaluation and its practice, and opportunities for reflection and learning in project development and post-delivery impact assessment. For “evaluation” and stems, interviewees stated that project evaluation in local governments is piecemeal, with a lack of clear objectives and trackable indicators. Learning opportunities from projects were felt to be truncated due to the lack of evaluation. Consultants working with local authorities can feel excluded from evaluation processes, despite often being heavily involved in project delivery.

*“Ongoing monitoring of targets or even having clear targets around some of our delivery is still currently missing” – Officer, Unitary Authority*

Other interviewees stated that a lack of cohesive planning, or a lack of strategic thinking, has led to project development and delivery being a “bitty” process, with projects set up to take advantage of funding opportunities, but with limited space for post-delivery learning. There was a clear desire from local authority officer interviews to track and measure progress more effectively.

For “reflection” and stems, one interviewee stated:

*“it's really interesting because it, this being forced to stop and think even if for an hour is really helpful” – Consultant, Tier 1 Local Authority*

This quote is indicative of the sentiment across the corpus for “reflection”, in that reflective space is powerful, not only for individual skills development but for project and process development in the future. The desire for “clear objectives, outcomes and trackable indicators” as one interviewee (Officer, Unitary Authority) stated is again present, but a counterpoint to this is senior-level buy-in and capacity to comprehend the importance of indicators, which interviewees were not confident of. The use of KPIs and outcome measures was questioned if the capacity to understand the data at a senior decision-maker level is not present. Interoperability of data sources, KPI measures and other indicators could mitigate this issue by offering a more standardised assessment framework, and comprehensible outputs for communication to non-expert decision-makers. Finally, the lack of a standardised national framework for evaluating the success or failure of energy-related projects, net zero projects and others was highlighted by interviewees: the importance of clarity from national government on measurement criteria for success was foregrounded.

## Discussion

Overall, there was limited evidence that evaluation practices were being undertaken by local authorities regarding net zero programs. However, this is not to say that evaluation was not desired: many respondents stated the importance of reflective periods towards the end of projects for enhancing future project development, or the desire for improved key performance indicators to evaluate the success or failure of projects. The question remains: what barriers to local authorities face in implementing evaluation practices, what steps can local authorities take in evaluating net zero or climate change projects more effectively, and what steps can be taken to ensure that key performance indicators or outcome measures are appropriate, informative and useful to senior decision-makers?

Regarding the thematic analysis of data availability and data interoperability, the core challenge is access to the “right” data sets for the focus of project development. Many respondents highlighted issues with previous or current projects where they felt stymied by a lack of availability of, or access to, appropriate data for evidence-based policy making. This can be because the data simply didn’t exist or hadn’t been collected, or there were barriers to data-sharing and collaboration. Internal data governance policies have been a blocker in some instances, leading to significant frustration for policy and delivery officers and elongated project delivery timeframes. Challenges remain in data maintenance also: clarity over who is responsible for maintaining organisational datasets or ensuring that external datasets are kept up to date is lacking. For effective evidence-based policy-making, the availability of accurate data is crucial, and local authorities are struggling to overcome this challenge. However, efforts to resolve this through online collaboration platforms and data platforms such as Net Zero Go are credited as having an impact on this issue for the local authorities involved in them.

From the wider thematic analysis, there remain a number of barriers to action in local governments on climate change. Two of these barriers were strongly represented both in the thematic analysis and content of the interviews: access to financial resources for projects, and access to human capital over time to develop and implement projects. The challenges of local authority finance in the UK are widely-discussed, but human resource constraints were an unexpected result of this work: local authorities feel challenged in staff capacity, not just in terms of time, but in terms of experience and long-term thinking. The “churn” of projects, as local authorities respond to short-term funding opportunities for short-term, low- to medium-scope projects, often leads to officers with significant experience in other fields being given more junior roles outside of their fields. One interviewee, as an example, was new in post as a Climate Change Officer, the sole member of their Climate Change Department, and was a career planner for local authorities. While there is transferable experience, deeper understanding of net zero concerns is being lost due to short-term outlooks. This has a cascading effect to project evaluation: the development of staff experience over time is compromised if the reassignment to new, short-term roles is continual, and as such the capacity to effectively evaluate projects from past experience is compromised. This is not the only way in which local authorities are losing experience over time: interviewees mentioned experiences where more-senior posts in climate change or net zero departments were replaced with lower-grade roles following staff retirements, or in some cases not replaced at all, degrading the capacity of the local authority in this area, but also reducing access to senior leadership and institutional knowledge.

Interviewees had a clear opinion on the role that the UK national government should take in net zero, being an enhanced policy framework for local authority climate action, further devolved powers, sufficient allocation of finance to develop and implement projects, and critically for this piece, clarity on measurement criteria and key performance indicators that constitute “success” in a given net zero project. This is a contributor to the current perception among the interviewees of the evaluation gap in local governments, the piecemeal nature of evaluation when it does happen, and the lack of capacity (both financial and human) dedicated to post-hoc learnings from project delivery. There is a need for greater clarity over what indicators should be used, what “success” in developing net zero projects represents, and what measures and indicators can be tracked over the project lifetime to ensure that this “success” is achieved.

While this research represents a sample of local authorities who have previously engaged, or are currently engaging with LAEPs, this is not exhaustive. Of the 19 respondents, this sample represents 14 local authorities in England (4.4% of the 317 total local authorities), with a further one each from Scotland and Wales, and no Northern Irish authorities, and as such will have inherent biases based on the respondents’ perceptions and experience. However, these interviews represent a sample of the different governance structures present in English local governments, being unitary authorities, county/district and borough (“two-tier”) authorities and combined authorities, and as such these insights are likely to be cross-applicable to different organisational structures of local governments in the United Kingdom.

## Conclusion

Achieving net zero by the legally-binding target date of 2050 is still possible in the United Kingdom, but will require significant enhancement of governance approaches at local, regional and national scales. Local authorities have a critical role to play in delivering net zero interventions, with deep place-based knowledge, understanding of local economies and the power of democratic mandates to enact change. However, local authorities are also compromised in intersecting ways, with financial pressures, the lack of staff capacity for project development and delivery, and challenges with post-delivery learning all contributing to a difficult delivery environment. The lack of statutory obligations for net zero adaptation, as well as a lack of mandated evaluation for net zero projects, and an uncoordinated national policy framework, present challenges for effective project development. This paper presents the outputs of semi-structured interviews with 19 respondents from 14 separate local authorities across England, Wales and Scotland, focusing on governance and delivery of net zero interventions. The lack of evaluation post-delivery of projects was evidenced through the interviews, and presents a clear risk to achieving net zero, with staff churn rates, time pressures and a lack of dedicated financing for post-hoc learnings meaning that outcomes are not being evaluated effectively, and potential future learnings for enhanced delivery are being missed. Data accessibility and interoperability will assist with this, but ensuring staff time is allocated to evaluation, and ensuring that resource is in place to allow for sufficient time to conduct evaluations, will help develop bodies of experience over time to enhance outcomes.

## Supplementary Information

Below is the link to the electronic supplementary material.

Supplementary file1 (DOCX 13 KB)

Supplementary file2 (DOCX 15 KB)

## Data Availability

No datasets were generated or analysed during the current study.

## Footnotes

1 For more information on English local authority structures, see How is local government organised? | Local Government Association.

2 The Local Energy Net Zero Accelerator programme is developing a set of geospatial mapping tools for strategic energy planning in local authorities (SSEN, 2025). The OnePlanet platform is a software tool to enable local governments and other organisations create sustainability action plans and strategic energy plans (OnePlanet, 2025). Net Zero Go is a project-focused software tool to aid local governments in developing project pipelines for net zero projects (Energy Systems Catapult, 2025).

## Declarations

### Clinical trial number

Clinical trial number: not applicable.
