# Supplementary material for: Governance for net zero project evaluation: Experiences from UK local authorities
Source: Energy Effic. 2026 Feb 24;19(3):20. doi: 10.1007/s12053-026-10422-9 (PMC12932288; doi:10.1007/s12053-026-10422-9)
Supplement: Supplementary file 1 — Supplementary file1 (DOCX 13 KB) [file 12053_2026_10422_MOESM1_ESM.docx]

**Supplementary Material A: Interview Schedule**

Initially, 9 questions were included in the interview schedule. Questions marked “a” were reserved for local authorities who were not currently engaging in Local Area Energy Plans:

1. Has your local authority had a Local Area Energy Plan commissioned, and if so, how long ago was the plan commissioned?

1a. If not, are you looking at commissioning a Local Area Energy Plan at present?

2. What progress has been made to date in implementing recommendations from the Local Area Energy Plan

2a. What stage is the Plan commissioning at in your local authority?

3. What are the barriers or enablers for the delivery of key actions identified under the LAEP for your area? These could be finance-related barriers, policy enablers, human resource-related challenges or similar.

4. How have local stakeholders influenced your decisions as to which aspects of the LAEP to take forward and in what order?

5. What types of stakeholders have you engaged with?

6. How do the barriers and enablers for the delivery of LAEP actions vary according to intervention area? For example, are transport-related barriers and enablers different to domestic energy barriers and enablers, and what common barriers are there?

3a. What barriers or enablers have you experienced for delivering climate change or net zero projects in your organisation? These could be finance-related barriers, policy enablers, human resource-related challenges or similar.

4a. How have stakeholder engagement practices influenced decision-making in your organisation with regard to climate change or net zero projects?

5a. What types of stakeholders have you consulted with?

6a. Do the barriers and enablers you described previously differ according to the area of intervention? For example, are transport-related barriers and enablers different to domestic energy barriers and enablers, and what common factors are there across intervention areas?

7. What do you feel are the most important governance-related factors for implementation of a LAEP across sectors? This could include clear policies, communication protocols and networking strategies, information-sharing strategies and implementation plans, and strong leadership and agenda-setting

8. What are the key governance-related enabling factors and challenges impacting the delivery of net zero interventions in your local area?

9. How do you feel the governance of net zero action planning and implementation could be improved in your area?

A tenth question was added to the interview schedule after the first three interviews were conducted, in response to an emerging topic of discussion:

10. Are local authorities the most appropriate delivery body for LAEP actions, and if not, who should be responsible for ensuring LAEP actions are delivered?
