# Supplementary material for: Governance for net zero project evaluation: Experiences from UK local authorities
Source: Energy Effic. 2026 Feb 24;19(3):20. doi: 10.1007/s12053-026-10422-9 (PMC12932288; doi:10.1007/s12053-026-10422-9)
Supplement: Supplementary file 2 — Supplementary file2 (DOCX 15 KB) [file 12053_2026_10422_MOESM2_ESM.docx]

**Supplementary Material B: Codebook and Definitions**

| **Top-Level Code** | **Sub-Codes** | **Description** |
| --- | --- | --- |
| Accountability and Ownership | Accountability | Accountability to self/others for ensuring net zero actions are delivered |
|  | Ownership of Role | Taking responsibility for ensuring net zero targets are acted upon and projects delivered |
| Resource and Capacity | Financial Resources | Financial capacity of Council to implement net zero projects, availability of internal/external funding |
|  | Human Resources | Staff capacity (time) for net zero actions |
|  | Technical Capacity | Engineering and technology understanding of responsible persons for net zero actions |
|  | Project Management Capacity | Ability of staff to conceptualise, develop and implement net zero projects |
| Communications and Information | Data Availability | Availability of data to support net zero decision making |
|  | Data Interoperability | Ability of data sources to be used by multiple organisations |
|  | Communications | Communication style and strategy for communicating net zero actions |
| Stakeholder Engagement | Public-Sector Stakeholders | Engagement with other public-sector bodies around net zero actions |
|  | Private-Sector Stakeholders | Engagement with commercial sector bodies and other private-sector organisations around net zero actions |
| Engagement w/ Neighbourhoods and Communities | Community Stakeholders | Engagement with communities and residents around net zero actions |
|  | Engagement Practices | Evidence of participatory development or lack thereof, engagement strategy and style |
| Coordination and Collaboration | Inter-Council Collaboration | Ability to work with District/Boroughs in two-tier authorities, other councils in unitaries/evidence thereof |
|  | Intra-Council Collaboration | Ability to work within own authority to develop projects and meet targets/evidence thereof |
|  | Governance Barriers | Barriers identified by interviewees relating to governance processes, be that timeframes, stringency or others |
|  | Coordinated Approaches | Evidence of multi-departmental working, efforts to reduce siloing |
| Future-Readiness | Responsiveness to Future Funding | Awareness of future funding resources or of ability to respond to changing market conditions |
|  | Responsiveness to Future Policy | Awareness of future policy developments and ability to respond |
|  | Behaviour Change | Understanding or awareness of the role of behaviour in net zero, encouragement of behavioural change |
| Fair, Equitable and Inclusive | Fairness, Equitability and Inclusivity | Evidence of energy justice principles, leave-no-one-behind principles, inclusivity in practice |
| LAEP Information | Critique of LAEP | Code for anything critiquing LAEP processes or outcomes |
|  | Details of LAEP | Information from Q1 on LAEPs |
|  | Progress Towards LAEP | Progress towards implementing recommendations from LAEPs |
| Leadership and Agenda-Setting | Senior-Level Buy-In | Engagement of senior leadership of Councils, commitment of senior leadership to net zero |
|  | Leadership | Demonstrable leadership in net zero sector, commitment to net zero of Council more widely |
| Project Delivery | Timeframes | Discussions around pace of change and project timelines, both development and delivery |
|  | Project Development | Information relating to development (specifically not delivery) of projects, project planning and related factors. |
|  | Skills and Supply Chain | Information relating to skills not captured by other codes, or local/national supply chains |
|  | Delivery Models | Information around different delivery models being used for intervention areas |
| Sentiment | Positive | Positive actions |
|  | Negative | Negative actions |
